# Biotransformation of 5-hydroxymethylfurfural into 2,5-dihydroxymethylfuran by *Ganoderma sessile* and toxicological assessment of both compounds

**DOI:** 10.1186/s13568-020-01023-5

**Published:** 2020-05-11

**Authors:** Ya-nan Hou, Ya-rong Wang, Chun-hui Zheng, Kun Feng

**Affiliations:** 1 Department of Bioengineering, Zhuhai Campus of Zunyi Medical University, Zhuhai, 519041 Guangdong China; 2 Biological Research and Development Centre, Zhuhai Campus of Zunyi Medical University, Zhuhai, 519041 Guangdong China; 3 Key Laboratory of Fundamental and Applied Research of Traditional Chinese Medicines, Zhuhai Campus of Zunyi Medical University, Zhuhai, 519041 Guangdong China

**Keywords:** *Ganoderma sessile*, Biotransformation, Antitumor, 5-hydroxymethylfurfural, 2,5-dihydroxymethylfuran

## Abstract

Biotransformation has the advantages of low cost and environmental protection and is a preferred method for production of compounds. At present, most 2,5-dihydroxymethylfuran (DHMF) is synthesized by chemical methods. In this study, 12.008 μg/mL DHMF was produced from 9.045 μg/mL 5-hydroxymethylfurfural (5-HMF) with a yield of 1.33 g/g using the crude enzymes from fungus *Ganoderma sessile*. To elucidate the toxic potential for both compounds, cytotoxicity tests and acute toxicity were evaluated respectively. 5-HMF induced weak cytotoxicity in HCT-8, A549 and SGC-7901 cells and DHMF exerted no cytotoxicity on HCT-8 while induced inhibition proliferation of A549 and SGC-7901 cells. The acute toxicity study showed no mortality happened in any group even at the single dose of 2000 mg/kg body weight. These results suggest it is feasible to convert 5-HMF to DHMF via crude enzymes from fungus *G. sessile* under mild condition, and that DHMF displays a potential effect of antitumor in vitro with little acute toxicity.

## Key points

1. 5-HMF was reduced to DHMF using crude enzymes from *G. sessile* with ratio of 1.33 g/g.
2. 5-HMF induced weak cytotoxicity on HCT-8, A549 and SGC-7901 cells.
3. DHMF showed cytotoxicity on A549 and SGC-7901 cells.
4. 5-HMF and DHMF displayed little acute toxicity.

## Introduction

*Codonopsis pilosula* and *Ganoderma sessile* are collectively regarded as famous herbal medicines and have been used in folk medicines for hundreds of years in China. Phytochemical researches show that *Ganoderma* sp. mainly contains triterpenoids (Zhao et al. 2011) and polysaccharides (Wang et al. 2019), which contribute to multiple bioactivities such as anti-tumor, anti-aging, antioxidant and immunomodulation (Yu et al. 2015). *G. sessile* is the most prevalent species in Eastern North America and is likely the species many of which are used medicinally in the United States. Meanwhile, some reports indicate that the main components of *C. pilosula* are polysaccharides (Liu et al. 2015), saponin (Jiang and Zhang 2011), and lobetyolin (Ma et al. 2014). Therefore, it was used to improve immunity (Zhao et al. 2013b), cure cancer (Chen et al. 2018), protect brain neurons (Hu and Chen 2015), and enhance the capacity of antioxidant (Liu et al. 2015).

*Codonopsis pilosula* is a Chinese herbal medicine that can be used for both medicine and food according to the Chinese Pharmacopoeia. As yet, there is no report on the toxicity of *C. pilosula.* However, there are many reports which have focused on 5-hydroxymethylfurfural (5-HMF), a major product of the Maillard reaction, can be extracted from *C. pilosula* (Feng et al. 2017; Li et al. 2009; Zhou et al. 2009). Some studies have reported that 5-HMF induces genotoxic (Fromowitz et al. 2012; Hoie et al. 2015), DNA-damaging (Pastoriza de la Cueva et al. 2016), cytotoxicity (Ji et al. 2018; Zhao et al. 2017), carcinogenesis (Florian et al. 2012) and mutagenicity (Hansruedi et al. 2012). Nonetheless, modern pharmacological studies have indicated that 5-HMF could exhibit numerous biological activities, such as antioxidant (Zhang et al. 2015; Zhao et al. 2013a), antiproliferative (Zhao et al. 2013a, 2014), anti-sickling (Galkin et al. 2015; Wright et al. 2015), and repairing cognitive impairment in Alzheimer’s disease (Liu et al. 2014). In addition, 5-HMF is one of the platform chemicals which can be transformed into various essential chemicals including 2,5-dihydroxymethylfuran (DHMF), 2,5-furandicarboxylic acid (FCDA) and 5-hydroxymethylfuroic acid (HMFCA) (Godan et al. 2019; Zhang et al. 2017). Interestingly, there is no report on the biological activity and safety of DHMF and this monomer is mostly synthesized by chemical reactions. Hence, the research on the biochemical synthesis of DHMF is meaningful to mechanism research, drugs development and clinical application. In fact, selective hydrogenation of 5-HMF and selective of catalysts are the key on the chemical synthesis of DHMF (Fulignati et al. 2019). Meanwhile, there are also studies showing that this transformation can be accomplished through biotransformation (He et al. 2018; Li et al. 2017).

Microbial transformation is the process by which fungus or bacteria are used to convert the substrate to a structurally related compound via one or a series of enzymatic reactions (Parshikov et al. 2012; Perkins et al. 2016). It is an excellent technique in modifying structure, enhancing efficacy and reducing toxicity of Traditional Chinese Medicine (TCM), due to its mild reaction conditions and convenient method (Cao et al. 2015; Xie et al. 2018). Hence, biotransformation plays an increasingly important role in chemistry.

This study presented the isolation and characterization of fractions with distinct variation between fermented *C. pilosula* with *G. sessile* (FCP) and non-fermented *C. pilosula* with *G. sessile* (NFCP). Furthermore, it was demonstrated that the variation was caused by enzymes from *G. sessile*. Subsequently, the cytotoxicity of 5-HMF and DHMF was evaluated with HCT-8, A549 and SGC-7901 cell lines. And the acute toxicity in rats treated with the above two compounds was investigated. To the best of our knowledge, this study is the first to explore a new method to generate DHMF with 5-HMF by *G. sessile* fermentation.

## Materials and methods

### Chemicals

5-HMF (CAS: 67-47-0, 97%) and DHMF (CAS: 1883-75-6, 98%) were purchased from MACKLIN (Shanghai, China). Acetonitrile (chromatographic grade) was obtained from EMD Millipore Corporation (Darmstadt, Germany). 3-(4,5-dimethyl-2-thiazolyl)-2,5-diphenyl-2-H-tetrazolium bromide (MTT) was obtained from Asegene (China). Cell counting kit-8 (CCK-8) supplied by DOJINDO (Japan). Roswell Park Memorial Institute 1640 (RPMI1640), Trypsin–EDTA, Fetal Bovine Serum (FBS) and Penicillin–Streptomycin Solution were obtained from Gibco (US). Phosphate Buffered Saline (PBS) was purchased from Hyclone (US). BCA Protein Assay Kit was obtained from Nanjing Jiancheng Bioengineering Institute (Nanjing, China).

Liquid medium was made of Mold Liquid Medium (Guangdong Huankai Microbial Sci. & Tech.co.,Ltd, China). Fermentation medium contained certain of concentration of the extracts from *C. pilosula* and inorganic salt ions according to the formula of Mold Liquid Medium. All other chemicals were of analytical grade.

### Preparation of extraction of *C. pilosula*

The radixes of *C. pilosula* were collected in Lanzhou, Gansu Province, China, and authenticated by Prof. Yang Chen from Zhuhai Campus of Zunyi Medical University. The dried roots (31.87 g) were extracted twice with boiling water, and it took 2 h for each extraction with material-liquid ratio of 1:16 (w/v). The rough extracts were decanted, filtered under vacuum to collect the supernatant. All the supernatant was merged to concentrate in a rotary evaporator (Hei-VAP, heidolph, Germany), and then dried (16.99 g) by freeze drying (2-4 LSC plus, CHRIST, Germany).

### Microorganism and fermentation

The fungus *G. sessile* (MUCL 38061) was deposited in Belgian Coordinated Collections of Microorganisms (308). Liquid medium was inoculated with the above fungus, and incubated for 7 days with 200 rpm at 28 °C. 20 g of *C. pilosula* extracts, 0.1 g of Magnesium sulphate and 0.2 g of Monopotassium phosphate were added to a 500 mL conical flask as a fermentation medium, and sterilized at 121 °C for 30 min after dissolved in 200 mL water. Then 20 mL mature liquid of fungus was added to cooling the fermentation medium, incubated for 7 days with 200 rpm at 28 °C to obtain FCP, and a same fermentation medium that injected 20 mL mature liquid of fungus was deposited at − 20 °C for 7 days server as NFCP. Subsequently their metabolites were centrifuged and analysed.

### Centrifugation and analysis by Ultra Performance Liquid Chromatography

The FCP and NFCP were centrifuged at 8000 rpm for 10 min. The samples which were prepared by the supernatant were passed through a filter (0.22 μm) before being injected into the Ultra Performance Liquid Chromatography (UPLC) system equipped with a Waters Photo-Diode Array (PDA) detector, Quaternary Solvent Manager (QSM) and ACQUITY UPLC^®^ BEH Shield RP18 (2.1 × 100 mm Colum, 1.7 μm). A gradient elution system made of solvent A (acetonitrile) and B (water) was used for the analysis and the routine was as follows: 0–4 min, 0–20% solvent A; 4–10 min, 20–100% solvent A; 10–12 min, 0–100% solvent B. The flow rate was 0.3 mL/min and the injection volume was 2 μL as well as the PDA detection wavelength ranging from 200 to 500 nm.

### Isolation and characterization by NMR and MS

The FCP and NFCP filtered through a 0.22 μm filter were chromatographically separated by preparative UPLC eluted with the mobile phase consisting of acetonitrile–water: 0–20 min, 0–20% solvent A; 20–50 min, 20–100% solvent A; 50–60 min, 0–100% solvent B, to afford compounds 1 and 2. Preparative UPLC analysis with UV detection at 284 nm and 224 nm was performed at a flow rate of 1.0 mL/min. The sample’s injection volume was 7 mL. The chromatographically separated liquid would be collected in stages for UPLC detection. The atmospheric pressure chemical ionization (APCI) data of two isolated compounds were collected using a Thermo Fisher Q Exactive. And ^1^H and ^13^C spectra were recorded with Bruker AVANCE III 500 MHz NMR Spectrometer with PABBO probe.

### Enzyme extraction from fermented *G. sessile*

The ectoenzyme was the supernatant (20 mL) collected from *G. sessile* fermentative liquid through vacuum filtering. And the procedure of the extraction of endoenzyme from mature fermented mycelia of *G. sessile* by ultrasound-assisted process was performed using an ultrasound device (PS-40T, JeKen, Dongguang, China) with frequency generator (40 kHz) according to the paper (Qin et al. 2016). In short, the same volume of distilled water (20 mL) was added as the ectoenzyme to mycelia collected by the below method in the 50 mL centrifuge tube. The mycelia were treated with Biospec Tissue-Tearor (Model 985370, USA) for 1 min, rested for 1 min, and repeated the steps above for 3 times. After that it would be treated for 1 min intermitted 1 min for 5 times with ultrasound device. Both of the procedures were operated in an ice water bath. After extraction, the crude extracts were filtered and then centrifuged at 5000 rpm for 10 min at 4 °C. The ectoenzyme and endoenzyme were stored at − 80 °C (Haier, China) for future usage.

### Biotransformation by enzyme

One unit (U) of enzymatic activity was defined as the total amount of the enzyme producing 1 μM of DHMF during 1 h. The BCA Protein Assay Kit was adopted to determinate protein contents of the enzyme mixture. And enzyme specific activity was calculated by the ration of enzymatic activity (U) to the concentration of protein (mg). Subsequently, a total of 1.0 mL solution system (pH 8.0) which includes 200 μL PBS, 1% (w/v) enzyme, 100 μL 5-HMF (100 μg/mL) and distilled water was incubated at 28 °C, 200 rpm for 48 h. The effect of biotransformation was measured by UPLC and the routine was same as above.

### The optimized biotransformation conditions

The effect of time, ectoenzyme amount and substrate amount on biotransformation was investigated through a total of 1.0 mL solution system (pH 8.0) containing PBS, 5-HMF solution, ectoenzyme and distilled water. And the concrete parameter was represented as Additional file 1: Table S1. Other conditions of biotransformation were 28 °C and 200 rpm. The production of DHMF was detected by UPLC and the routine was same as above.

### Cell culture and cytotoxicity test

Cell lines HCT-8 (3111C0001CCC000104) and A549 (3131C0001000700150) were obtained from National Infrastructure of Cell Line Resource (Beijing, China). SGC-7901 cell line was purchased from Suer Shengwu (Shanghai, China). All cells maintained in a humidified atmosphere at 37 °C and 5% CO_2_ in RPMI 1640 supplemented with 10% FBS and 1% Penicillin–Streptomycin solution. Cells were seeded in 96-well plates at a density of 8 * 10^3^ cells per well and then incubated with various concentrations of 5-HMF and DHMF (Additional file 2: Table S2) for 24 h, respectively. Subsequently, culture supernatant was removed. HCT-8 cells were incubated in 100 μL fresh basal medium and 10 μL CCK-8 solution at 37 ℃ for 2 h. The optical density (OD) value of each well was measured at 450 nm using spectrophotometer (Multiskan Go 1510, Thermo, US); A549 and SGC-7901 cells were incubated in 100 μL MTT solution (1 mg/mL) at 37 ℃ for 4 h. Subsequently, the liquid was removed and dissolved the formazan with 150 μL DMSO, and the OD value was measured at 490 nm. Cell viability was given in a percentage of the control group. All data repeated for three times.

### Animals

Female adult healthy Sprague–Dawley (SCXK(Lu) 2014 0007) rats (200 ± 20 g) were purchased from Jinan Pengyue Experimental Animal Breeding Co., Ltd. The rats were adaptive housed in cage for 5 days before the formal experiment. The temperature in the experimental animal room was 22 ± 1 °C and the relative humidity was 60% ± 5%, additionally, lighting conditions was 12-h light/dark cycle.

### Acute toxicity assessment

Acute toxicity assessment was carried out in accordance with the Organization for Economic Corporation and Development (OECD) guidelines (Section 4, number-423, Acute Oral Toxicity-Acute Toxicity Class Method) (OECD 2002). Twenty-four rats were randomly divided into five groups. Six rats were in the control group as well as the high-dose groups, and three rats were in the low-dose groups. Before the experiment, all rats needed to be fastened but water was withheld overnight. Briefly, the control group received distilled water while experimental groups were orally treated with test compounds at a single dose of 300 mg/kg 5-HMF, 300 mg/kg DHMF, 2000 mg/kg 5-HMF, and 2000 mg/kg DHMF, respectively. The animals were monitored periodically during the first 24 h, especially the opening 30 min and 4 h, after drug oral treatment for any signs of toxicity. The rats were further observed for 14 days. Moreover, the body weight was measured at the first and last day. At the end of experiment, all rats were anaesthetized and their brains, hearts, lungs, livers, kidneys, and spleens were collected, weighed and observed macroscopically.

### Statistical analysis

The data were showed as mean ± standard deviation (SD). The statistical analysis was performed using one-way ANOVA and one-way repeated measures ANOVA. Values of *P *< 0.05 were counted as statistically significance. The applied software was GraphPad Prism 5.0 and SPSS 21.0 version.

## Results

### UPLC analysis of composition changes after fermentation

**Fig. 1:**
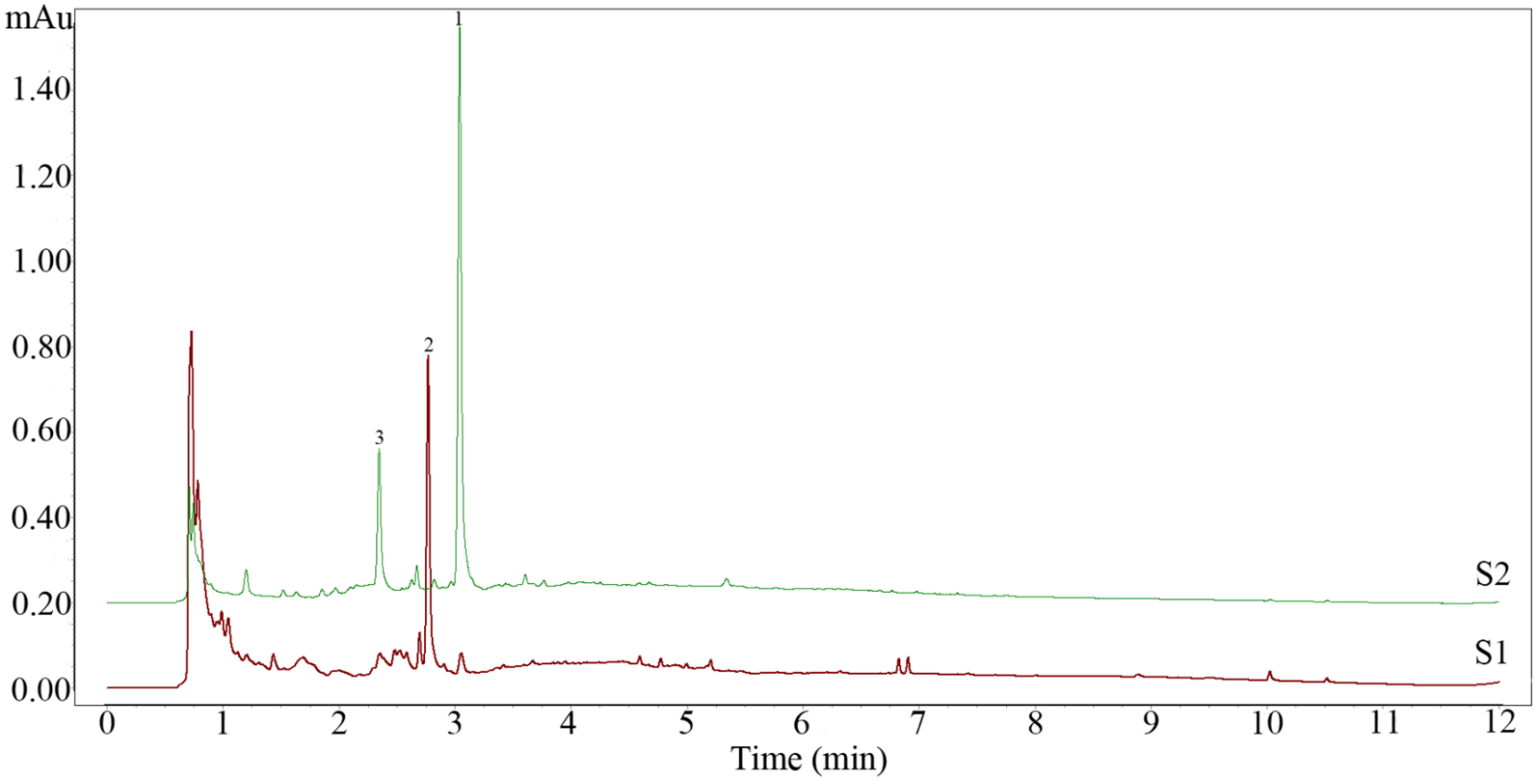
UPLC chromatograms of the NFCP (S2) and FCP (S1). NFCP: non-fermented *C. pilosula* with *G. sessile*; FCP: fermented *C. pilosula* with *G. sessile*

Shown as Fig. 1, the peak value of numbered 1 was 1.35 mAu with retention time of 3.08 min, however, the peak of it sharply decreased to 0.06 mAu and peak 2 emerged after fermentation. The peak value of numbered 2 was 0.77 mAu with retention time of 2.81 min. Moreover, peak 3 was disappeared completely after fermentation. This experiment confirmed the conversion of compound 1 to compound 2 during fermentation.

### Preparative UPLC chromatographic separation and identification of compounds

**Fig. 2:**
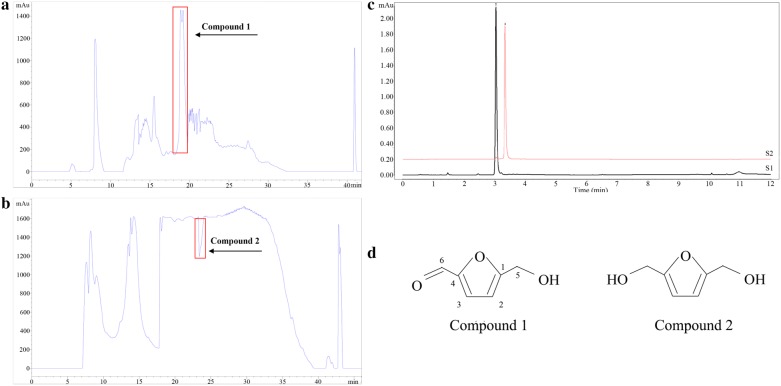
Preparative UPLC chromatograms of two compounds and its structures. Compound 1 separated from NFCP (**a**) and detection wavelength at 284.9 nm, compound 2 separated from FCP (**b**) and detection wavelength at 224.6 nm. (↑) means the target segment. Purified substances were represented as the UPLC chromatograms (**c**). S1 represented compound 1, S2 represented compound 2. The structures of purified substances were shown as (**d**)

The isolation of targeted ingredients was performed through preparative UPLC. The NFCP and FCP were soluble in water and subjected to preparative UPLC to separate and purify. The peak value of compound 1 (Fig. 2a) was 1500 mAu with the retention time of 18.6–20.1 min, while compound 2 (Fig. 2b) separated within 23.0–24.1 min and formed a negative peak. As a result, two compounds isolated successfully were distinctly detected by UPLC shown in the Fig. 2c: compound 1 (characteristic wavelength was 284.9 nm, S2) and compound 2 (characteristic wavelength was 224.6 nm, S1). The purity of these compounds was identified by UPLC. And the results showed that the purity of compound 1 was 96.51% and 92.84% for compound 2. Subsequently, they were identified as 5-HMF and DHMF via spectral detection.

5-HMF (compound 1): brown oily liquid; ESI–MS: m/z 127 [M+Na]+; 1H NMR (400 MHz, DMSO-d6) δ: 9.55 (s, 1H, H-6), 7.50 (d, J = 3.5 Hz, 1H, H-3), 6.61 (d, J = 3.5 Hz, 1H, H-2), 4.51 (s, 2H, H-5); 13C NMR (126 MHz, DMSO-d6) δ: 178.44 (s, C-6), 162.64 (s, C-1), 152.19 (s, C-4), 124.91 (s, C-3), 110.15 (s, C-2), 56.40 (s, C-5). All data was in accordance with reference (Serra Cayuela et al. 2013).

DHMF (compound 2): yellow powder; ESI–MS: m/z 129 [M+H]+; 1H NMR (500 MHz, DMSO-d6) δ: 6.18 (s, 2H, H-2, H-3), 4.35 (s, 4H, H-5, H-6); 13C NMR (126 MHz, DMSO-d6) δ: 155.08 (s, C-1), 107.84 (s, C-2), 107.84 (s, C-3), 155.08 (s,C-4), 56.16 (s, C-5), 56.16 (s, C-6). All data were in accordance with reference (Goswami et al. 2008). The chemical structure of two compounds was presented in Fig. 2d.

### The effects of biotransformation

**Fig. 3:**
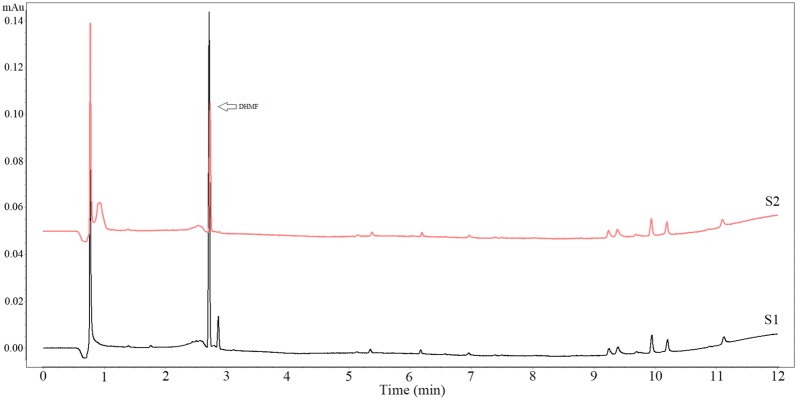
The effect of endoenzyme and ectoenzyme on biotransformation of 5-HMF. The PBS volume, time of biotransformation, temperature and initial pH were set as 200 μL, 48 h, 28 °C and pH neutral. λ = 224.6 nm. S1 represented ectoenzyme and S2 represented endoenzyme

**Fig. 4:**
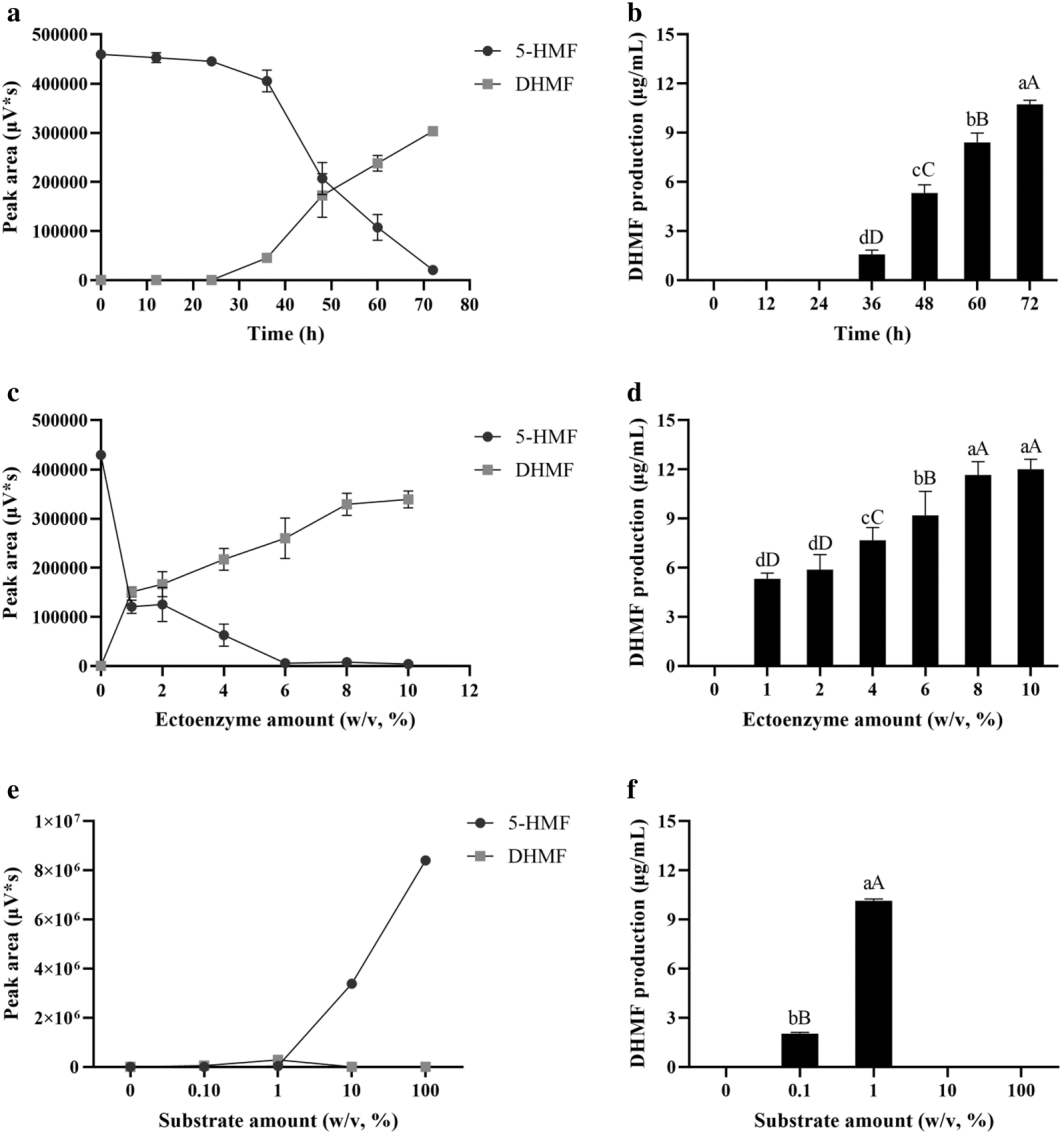
The effect of time (**a**, **b**), ectoenzyme amount (**c**, **d**) and substrate amount (**e**, **f**) on biotransformation of 5-HMF. Data were represented as mean ± SD and the experiment was repeated three times. Columns labelled with different lower case letters were significantly different at *P *< 0.05, and upper case letters were significantly different at *P *< 0.01 (S–N–K). For example, there was a significant difference in the biotransformation rates between 72 and 60 h (*P *< 0.01)

The activity of endoenzyme was 4716.98 U while the activity of ectoenzyme was 5970.15 U/mg. The activity of ectoenzyme was much higher than that of endoenzyme (*P *< 0.05). The effect of ectoenzyme on biotransformation was better than that in endoenzyme shown as Fig. 3. The production of DHMF at different time was determined and the production of DHMF was positively correlated with the biotransformation time (Fig. 4a, b). And the results showed that 8% of the enzyme amount exhibited the same bioconversion performance as the enzyme amount at 10% (Fig. 4c, d). Based on the optimization studies performed on ectoenzyme, an incubation temperature of 28 °C, enzyme amount at 8% (w/v), substrate amount at 1% (w/v), initial pH of solution system at 8.0, and incubation time of 72 h, a complete catalytic reduce of 5-HMF to DHMF was achieved at a yield of 1.12 g/g (Fig. 4e, f). However, the maxim yield was 1.33 g/g at the 10% enzyme amount and 1% substrate amount (Fig. 4d) in the biotransformation.

### Cytotoxicity assay

**Fig. 5:**
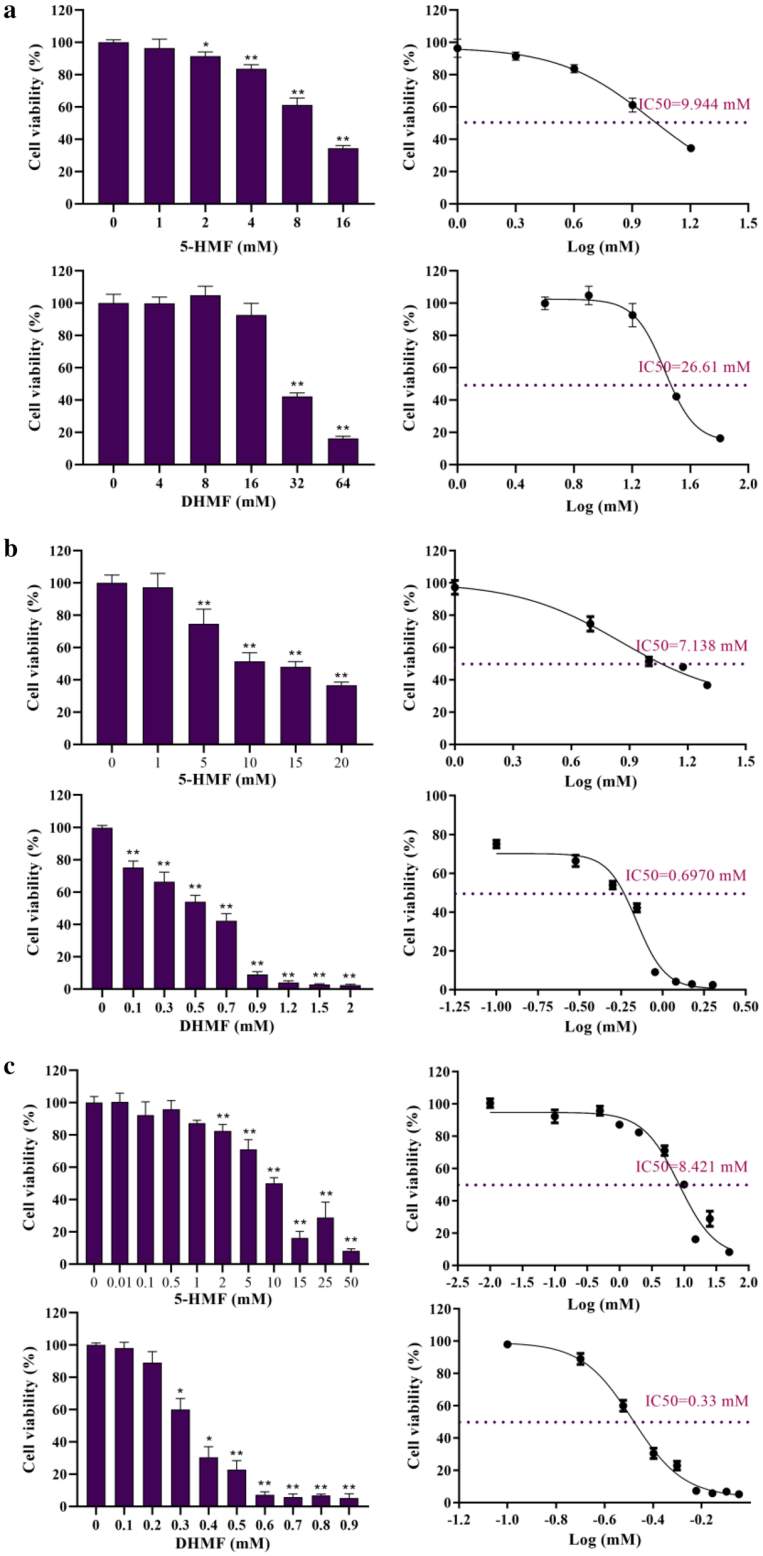
Cytotoxicity of 5-HMF and DHMF. Data were represented as mean ± SD and the experiment was repeated three times. **a** Referred to the cytotoxicity of compounds on HCT-8 cells, **b** referred to the cytotoxicity of compounds on A549 cells, **c** referred to the cytotoxicity of compounds on SGC-7901 cells. IC_50_ value was analysed by GraphPad Prism

The cytotoxicity was expressed as IC_50_ value, concentration at which 50% of the cells were survived in RPMI 1640. HCT-8 cells with 5-HMF and DHMF resulted in IC_50_ value of 9.944 mM and 26.61 mM, respectively (Fig. 5a). As for A549 cells, the IC_50_ value of 7.138 mM was determined for 5-HMF; under the same conditions the IC_50_ value of DHMF was 0.697 mM (Fig. 5b). Moreover, for SGC-7901 cells, the IC_50_ value was 8.421 mM of 5-HMF while 0.33 mM of DHMF (Fig. 5c).

### Acute toxicity study

**Fig. 6:**
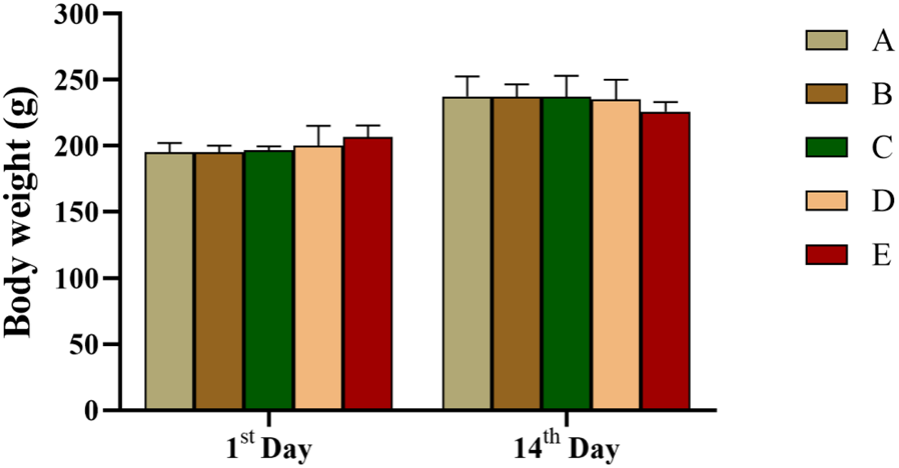
Changes in body weight after administration in rats. Values represented as mean ± SD and analyzed by one-way repeated measures ANOVA test. No letters in the columns means *P *> 0.05 vs. control group, *n *= 6 of A, D and E groups, *n *= 3 of B and C groups. A: Distilled water (0 mg/kg); B: 5-HMF (300 mg/kg); C: DHMF (300 mg/kg); D: 5-HMF (2000 mg/kg); E: DHMF (2000 mg/kg)

In the acute toxicity study, none of the rats died during the 14-day period of monitor treated with 5-HMF or DHMF. However, there were some slight abnormal changes in their behaviour that related to the nervous system (Table 1). The body weight of the tested groups was similar to the control group (Fig. 6). And the weight of organs including brain, heart, lung, liver, kidney and spleen of rats treated with compounds, was not remarkably different compared to the control group (Table 2). Similarly, there was no abnormality in the morphology of organs. The pathological sections also had no difference (Additional file 3: Figure S1). From the perspective of behavioural performance, the toxicity of 5-HMF was higher than DHMF, but stayed within the normal reference range. According to annex 2c: Test procedure with a starting dose of 300 mg/kg body weight (OECD 2002), LD50 cut-off of rats treated with 5-HMF and DHMF were greater than 2000 mg/kg B.W.

**Table 1:** Abnormal performance in behavioristics

Groups	Abnormal behaviours
A	None
B	During the first 30 min, all rats became quitter and slightly weaker than the control, and returned to normal within 1 h
C	During the first 30 min, all rats became quitter and slightly weaker than the control, and returned to normal within 1 h
D	Rat numbered 1 emerged convulsions, lethargy, convulsions, and limb weakness during the first 30 min, showed somnolence and decrease of animal heat in the next 30 min; others only behaved a quiet symptom, all rats returned to normal within 1 h
E	In the first 30 min, all rats were more active than control, then showed lethargic and dilatoriness, yet returned to normal after 30 min

Tables denoted the abnormal performance in behavioristics during the first 4 h of continuous observation, *n *= 6 of A, D and E groups, *n *= 3 of B and C groups. A: Distilled water (0 mg/kg); B: 5-HMF (300 mg/kg); C: DHMF (300 mg/kg); D: 5-HMF (2000 mg/kg); E: DHMF (2000 mg/kg)

**Table 2:** Organs index of rats treated with 5-HMF and DHMF (g/100 g of body weight)

Organs	A	B	C	D	E
Liver	3.12 ± 0.31	3.06 ± 0.19	3.04 ± 0.26	2.83 ± 0.09	3.05 ± 0.17
Spleen	0.22 ± 0.01	0.25 ± 0.01	0.22 ± 0.04	0.25 ± 0.06	0.22 ± 0.02
Kidney	0.73 ± 0.06	0.75 ± 0.06	0.75 ± 0.07	0.75 ± 0.07	0.75 ± 0.06
Lung	0.50 ± 0.02	0.52 ± 0.02	0.47 ± 0.01	0.51 ± 0.04	0.49 ± 0.03
Heart	0.34 ± 0.01	0.35 ± 0.03	0.32 ± 0.02	0.33 ± 0.02	0.32 ± 0.04
Brain	0.79 ± 0.05	0.74 ± 0.02	0.68 ± 0.09	0.74 ± 0.05	0.79 ± 0.07

Values represented as mean ± SD and analyzed by one-way ANOVA with S–N–K test. *P *> 0.05 vs. control group, *n *= 6 of A, D and E groups, *n *= 3 of B and C groups. A: Distilled water (0 mg/kg); B: 5-HMF (300 mg/kg); C: DHMF (300 mg/kg); D: 5-HMF (2000 mg/kg); E: DHMF (2000 mg/kg)

## Discussions

That fermentation of *Codonopsis* plays a role of improving memory impairment, neuroprotective and anti-tumour have been investigated and reported (Lee et al. 2015; Weon et al. 2014, 2016). However, most of the existing reports are about *Codonopsis lanceolata*, and there is no precedent for *C. pilosula* fermented by *G. sessile*. In this research, significant changes between NFCP and FCP were discovered as anticipating. As one of the microbial biotransformation technologies, fermentation is often utilized to modify chemical structure owing to highly reaction specific, enantiomer-specific and region-specific (Hegazy et al. 2015). Hence, there is no wonder of the occurrence of such distinction.

This research worked on the isolation of targeted ingredients through preparative UPLC. Subsequently, they were identified as 5-HMF and DHMF via spectral detection. It has been reported that 5-HMF was found in *Codonopsis* (Dou et al. 2017). These results implied a method of producing DHMF under mild reaction conditions and convenient operation. In fact, DHMF is a downstream product of 5-HMF, and their structural difference only lies in the aldehyde and hydroxyl on the side chain of the furan ring. Moreover, the major approach to obtain DHMF was chemical synthesis that required harsh reaction conditions. For example, apart from catalyst composed of a rare element and reaction temperature over 100 °C (Qin et al. 2016; Upare et al. 2018). It is an important pathway that catalytic transfer hydrogenation of 5-HMF into DHMF via Meerwein–Ponndorf–Verley reaction. However, the premise of that is the corresponding catalyst (Hu et al. 2018).

Microorganisms are capable to produce a multiple of enzymes to catalyse basic chemistry reactions including redox reaction (Perkins et al. 2016). For example, *O*-benzoquinone have been produced by reacting laccase oxidized Catechol (Xu et al. 2016). Previous studies have demonstrated that biocatalytic reduction of 5-HMF to DHMF using whole resting cells through a new highly tolerant yeast strain-*Meyerozyma guilliermondii* SC1103 with a yield of 86% (Li et al. 2017). Moreover, DHMF was biologically synthesized from 5-HMF with *E. coli* CCZU-K14 cells (He et al. 2018). Given that the biocatalysis might come from relevant enzyme of *G. sessile*, the crude enzymes were extracted from fermented *G. sessile* and applied to biotransform 5-HMF. As a result, it is demonstrated that 5-HMF becomes DHMF through the enzyme, and the biotransformation rate was as high as 1.33 g/g.

Taking the controversial safety of 5-HMF (Abraham et al. 2011; Severin et al. 2010) and unclear toxicity of DHMF into consideration, compounds 5-HMF and DHMF were evaluated for their cytotoxicity against HCT-8, A549 and SGC-7901 cell lines as well as acute toxicity via MTT and CCK-8 method. The results can be concluded that DHMF is safer than 5-HMF in HCT-8 cell, whereas shows greater toxicity in A549 and SGC-7901 cell lines. That different cell lines have different levels of sensitivity may account for the discrepancy. Nevertheless, the IC_50_ of 5-HMF was not more than the threshold of producing serious toxicity which was consistent with the report of none cytotoxic in human skin fibroblast cells treated with 5-HMF (Frade et al. 2014). It is worth noting that DHMF exhibited severe cytotoxicity on A549 and SGC-7901 cells which indicated that it has potential biological activity of antitumor, but its mechanism still needs further research.

In this acute toxicity assessment, only very slightly abnormal behaviours occurred in the low-dose group. Considering using the least amount of the animals, only three rats were used in these groups. And behaviouristics as well as the weight of organs and pathology of female rats treated by 5-HMF and DHMF were investigated, respectively. The results revealed that neither the 5-HMF nor DHMF are capable of leading to death even at the dose of 2000 mg/kg. Previous studies have also indicated that no adverse effect in terms of weight growth, food and water intake, and histological examination of organs regarding acute toxicity in rats (Abraham et al. 2011). While other studies showed that mild nephrotoxic effects are detected in mice at the dose up to 536 mg/kg and exposed to 5-HMF in drinking water for 12 weeks (Bauer Marinovic et al. 2012). As a consequence, all the evidence is inclined to the fact that the 5-HMF absorbed by daily life is not enough. In contrast to the 5-HMF, the DHMF showed less toxicity only in behaviouristics and no statistical difference in other indicators, suggesting that DHMF is safer than 5-HMF which happen to coincide with the result of cytotoxicity. Therefore, the study demonstrated it is feasible that 5-HMF can be converted to DHMF via crude enzyme from fungus *G. sessile* under mild condition, and 5-HMF showed low toxicity in vitro or in vivo. However, DHMF displays potential effect of antitumor in vitro with little acute toxicity.

## Supplementary information

**Additional file 1: Table S1.** The concrete parameter of optimized biotransformation.

**Additional file 2: Table S2.** Final concentration of drugs used in cells.

**Additional file 3: Figure S1.** Histopathological analysis of the liver, kidney and spleen (haematoxylin/eosin, 200×) of the rats treated with single dose of 5-HMF or DHMF. Liver sections of all groups showed normal hepatic cells with well-preserved cell structure. Kidney sections of all groups showed renal tissue with normal glomeruli. Spleen section of all groups showed normal splenic architecture with normal lymphoid follicles.

## Data Availability

The datasets generated during and/or analysed during the current study are available from the corresponding author on reasonable request.
