# Supplementary material for: Biotransformation of 5-hydroxymethylfurfural into 2,5-dihydroxymethylfuran by Ganoderma sessile and toxicological assessment of both compounds
Source: AMB Express. 2020 May 11;10:88. doi: 10.1186/s13568-020-01023-5 (PMC7214591; doi:10.1186/s13568-020-01023-5)
Supplement: Supplementary file 1 — Additional file 1: Table S1. The concrete parameter of optimized biotransformation. [file 13568_2020_1023_MOESM1_ESM.docx]

***AMB Express***

**Biotransformation of 5-hydroxymethylfurfural into 2,5-dihydroxymethylfuran by *Ganoderma sessile* and** **toxicological assessment of both compounds**

Ya-nan Hou^1#^, Ya-rong Wang^2#^, Chun-hui Zheng^1#^, Kun Feng^1,3^*

^1^ Department of Bioengineering, Zhuhai Campus of Zunyi Medical University, Zhuhai 519041, Guangdong, China.

^2^ Biological Research and Development Centre, Zhuhai Campus of Zunyi Medical University, Zhuhai 519041, Guangdong, China.

^3^ Key Laboratory of Fundamental and Applied Research of Traditional Chinese Medicines, Zhuhai Campus of Zunyi Medical University, Zhuhai 519041, Guangdong, China.

^#^ These authors contributed equally to this work.

***Corresponding author.** E-mail: fengk@zmu.edu.cn

**Table S1** The concrete parameter of optimized biotransformation

| Optimized conditions | PBS (μL) | 5-HMF (w/v, %) | Ectoenzyme amount (w/v, %) | Time (h) |
| --- | --- | --- | --- | --- |
| Time | 200 | 1 | 5 | 0, 12, 24, 36, 48, 60, 72 |
| Ectoenzyme amount | 200 | 1 | 0, 2, 4, 6, 8, 10 | 72 |
| Substrate amount | 200 | 0, 0.1, 1, 10, 100 | 8 | 72 |
