# Supplementary material for: Biotransformation of 5-hydroxymethylfurfural into 2,5-dihydroxymethylfuran by Ganoderma sessile and toxicological assessment of both compounds
Source: AMB Express. 2020 May 11;10:88. doi: 10.1186/s13568-020-01023-5 (PMC7214591; doi:10.1186/s13568-020-01023-5)
Supplement: Supplementary file 2 — Additional file 2: Table S2. Final concentration of drugs used in cells. [file 13568_2020_1023_MOESM2_ESM.docx]

***AMB Express***

**Biotransformation of 5-hydroxymethylfurfural into 2,5-dihydroxymethylfuran by *Ganoderma sessile* and** **toxicological assessment of both compounds**

Ya-nan Hou^1#^, Ya-rong Wang^2#^, Chun-hui Zheng^1#^, Kun Feng^1,3^*

^1^ Department of Bioengineering, Zhuhai Campus of Zunyi Medical University, Zhuhai 519041, Guangdong, China.

^2^ Biological Research and Development Centre, Zhuhai Campus of Zunyi Medical University, Zhuhai 519041, Guangdong, China.

^3^ Key Laboratory of Fundamental and Applied Research of Traditional Chinese Medicines, Zhuhai Campus of Zunyi Medical University, Zhuhai 519041, Guangdong, China.

^#^ These authors contributed equally to this work.

***Corresponding author.** E-mail: fengk@zmu.edu.cn

**Table S2** Final concentration of drugs used in cells

| Cells | Concentration (mM) | |
| --- | --- | --- |
|  | 5-HMF | DHMF |
| HCT-8 | 0, 1, 2, 4, 8, 16 | 0, 4, 8, 16, 32, 64 |
| A549 | 0, 1, 5, 10, 15, 20 | 0, 0.1, 0.3, 0.5, 0.7, 0.9, 1.2, 1.5, 2 |
| SGC-7901 | 0, 0.01, 0.1, 0.5, 1, 2, 5, 10, 15, 25, 50 | 0, 0.1, 0.2, 0.3, 0.4, 0.5, 0.6, 0.7, 0.8, 0.9 |
