# Supplementary material for: Biotransformation of 5-hydroxymethylfurfural into 2,5-dihydroxymethylfuran by Ganoderma sessile and toxicological assessment of both compounds
Source: AMB Express. 2020 May 11;10:88. doi: 10.1186/s13568-020-01023-5 (PMC7214591; doi:10.1186/s13568-020-01023-5)
Supplement: Supplementary file 3 — Additional file 3: Figure S1. Histopathological analysis of the liver, kidney and spleen (haematoxylin/eosin, 200×) of the rats treated with single dose of 5-HMF or DHMF. Liver sections of all groups showed normal hepatic cells with well-preserved cell structure. Kidney sections of all groups showed renal tissue with normal glomeruli. Spleen section of all groups showed normal splenic architecture with normal lymphoid follicles. [file 13568_2020_1023_MOESM3_ESM.pdf]

AMB Express

Biotransformation of 5-hydroxymethylfurfural into 2,5-dihydroxymethylfuran by *Ganoderma sessile* and toxicological assessment of both compounds

Ya-nan Hou[1#], Ya-rong Wang[2#], Chun-hui Zheng[1#], Kun Feng[1,3]*

[1] Department of Bioengineering, Zhuhai Campus of Zunyi Medical University, Zhuhai 519041, Guangdong, China.

[2] Biological Research and Development Centre, Zhuhai Campus of Zunyi Medical University, Zhuhai 519041, Guangdong, China.

[3] Key Laboratory of Fundamental and Applied Research of Traditional Chinese Medicines, Zhuhai Campus of Zunyi Medical University, Zhuhai 519041, Guangdong, China.

[#] These authors contributed equally to this work.

***Corresponding author.** E-mail: fengk@zmu.edu.cn

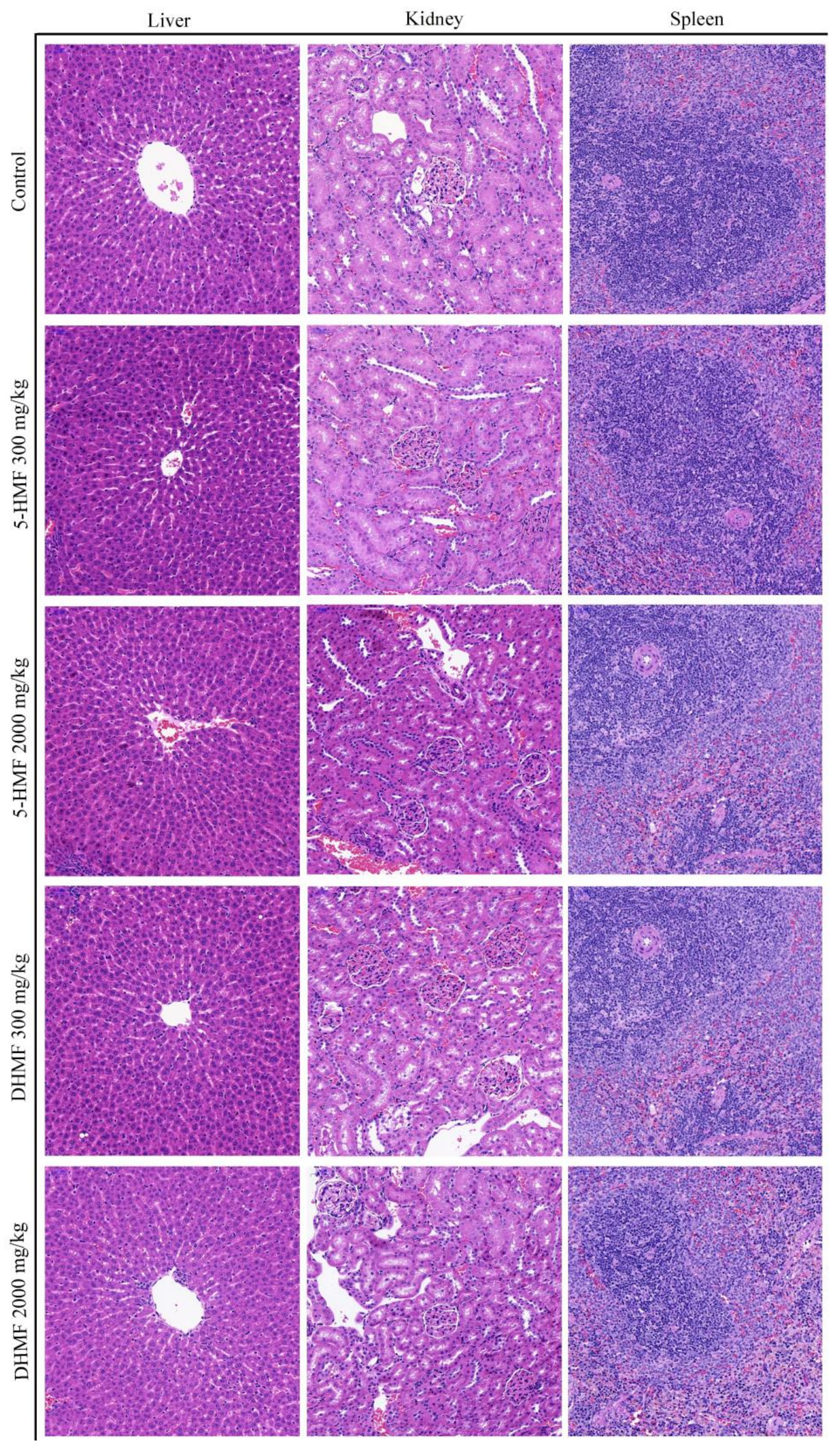

Figure S1 Histopathological analysis of the liver, kidney and spleen (haematoxylin/eosin, 200X) of the rats treated with single dose of 5-HMF or DHMF

Liver sections of all groups showed normal hepatic cells with well-preserved cell structure. Kidney sections of all groups showed renal tissue with normal glomeruli. Spleen section of all groups showed normal splenic architecture with normal lymphoid follicles.
